# 

**Published:** 1900-01-20

**Authors:** 


### THE HOSPITAL

**CADBURY'S COCOA** NO DRUGS OR ALKALIES USED.

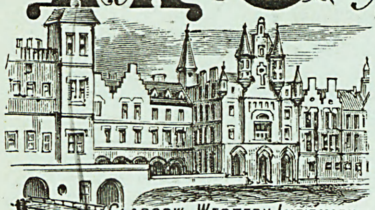

GLASGOW WESTERN INFIRMARY

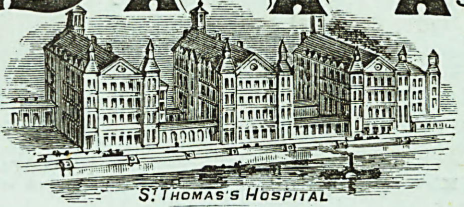

ST. THOMAS'S HOSPITAL

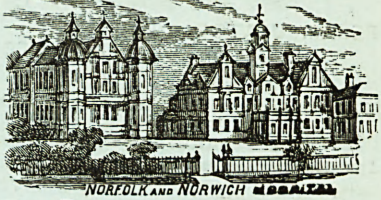

NORFOLK AND NORWICH HOSPITAL

No. 695. Vol. XXVII. [Registered as a Newspaper.] SATURDAY, JAN. 20, 1900. [Subscription Terms, see p. 254.] **PRICE TWOPENCE.**

